# Role of *Trichoderma reesei* mitogen-activated protein kinases (MAPKs) in cellulase formation

**DOI:** 10.1186/s13068-017-0789-x

**Published:** 2017-04-20

**Authors:** Mingyu Wang, Meiling Zhang, Ling Li, Yanmei Dong, Yi Jiang, Kuimei Liu, Ruiqin Zhang, Baojie Jiang, Kangle Niu, Xu Fang

**Affiliations:** State Key Laboratory of Microbial Technology, School of Life Sciences, Shandong University, Jinan, China

**Keywords:** *Trichoderma reesei*, Cellulase, MAP kinase, Signal transduction, Cell wall integrity, Transcriptional analysis

## Abstract

**Background:**

Despite being the most important cellulase producer, the cellulase-regulating carbon source signal transduction processes in *Trichoderma reesei* are largely unknown. Elucidating these processes is the key for unveiling how external carbon sources regulate cellulase formation, and ultimately for the improvement of cellulase production and biofuel production from lignocellulose.

**Results:**

In this work, the role of the mitogen-activated protein kinase (MAPK) signal transduction pathways on cellulase formation was investigated. The deletion of yeast *FUS3*-like *tmk1* in *T. reesei* leads to improved growth and significantly improved cellulase formation. However, *tmk1* deletion has no effect on the transcription of cellulase-coding genes. The involvement of the cell wall integrity maintenance governing yeast Slt2-like Tmk2 in cellulase formation was investigated by overexpressing *tmk3* in *T. reesei Δtmk2* to restore cell wall integrity. Transcriptional analysis found little changes in cellulase-coding genes between *T. reesei* parent, *Δtmk2*, and *Δtmk2::OEtmk3* strains. Cell wall integrity decreased in *T. reesei Δtmk2* over the parent strain and restored in *Δtmk2::OEtmk3*. Meanwhile, cellulase formation is increased in *T. reesei Δtmk2* and then decreased in *T. reesei Δtmk2::OEtmk3.*

**Conclusions:**

These investigations elucidate the role of Tmk1 and Tmk2 on cellulase formation: they repress cellulase formation, respectively, by repressing growth and maintaining cell wall integrity, while neither MAPK regulates the transcription of cellulase-coding genes. This work, together with the previous investigations, suggests that all MAPKs are involved in cellulase formation, while Tmk3 is the only MAPK involved in signal transduction for the regulation of cellulase expression on the transcriptional level.

**Electronic supplementary material:**

The online version of this article (doi:10.1186/s13068-017-0789-x) contains supplementary material, which is available to authorized users.

## Background

The economical and environment-friendly utilization of earth’s largest biomass reserve lignocellulose for the purpose of biofuel production requires the abundant production of cellulase, a multicomponent synergistic enzyme cocktail secreted by microorganisms [1]. *Trichoderma reesei* (syn. *Hypocrea jecorina*) is the most prominent industrial cellulase producer that was demonstrated capable of producing as much as 20 g L^−1^ cellulases [2]. This filamentous fungus has therefore been considered a model organism for cellulase formation and regulation mechanisms [3].

The formation of cellulases has been shown to be controlled on both transcriptional and secretion levels in filamentous fungi [4, 5]. Transcriptionally, cellulase-coding genes are co-regulated by external signals such as carbon signals (such as glucose, cellobiose, lactose, Avicel, and sophorose) and light signals [6–9]. Of these signals, glucose is a strong inhibitory signal, while Avicel, lactose, sophorose, cellobiose, and light signals are inducing signals for the expression of cellulase-coding genes. Transcription factors that govern the stimulation or inhibition of cellulase-coding genes have been investigated in great detail, leading to the identification of inhibitory transcription factors Cre1 and ACEI [10, 11], as well as activating transcription factors Xyr1 and ACEII [12, 13]. However, how signals are passed from extracellular space to transcription factors for the modulation of cellulase expression is largely unclear.

In the past decade, the most significant breakthrough on the understanding of cellulase-regulating signal transduction pathways is the elucidation of the light regulation pathway [9]. This signal transduction is executed by a complex system in which the second messenger cyclic AMP (cAMP) and protein kinase A (PKA) play a central role [14]. The understanding of the carbon signal transduction pathways, however, is still preliminary. Previous bioinformatic analysis suggested that cellulase-regulating transcription factors Cre1, Xyr1, ACEI, and ACEII all harbor phosphorylation sites for protein kinases such as casein kinase II (CKII) and mitogen-activated protein kinases (MAPKs) [15]. It was also shown that the disruption of CKII phosphorylation site on Cre1 leads to Cre1’s loss of phosphorylation and subsequently loss of glucose inhibition [16]. Combined with the findings that protein kinases such as Ime2, Sch9, and Yak1 regulate cellulase formation [17, 18], these results strongly suggest protein kinases may play an important role in relaying cellulase-regulating signals. Of these protein kinases, the most interesting protein kinases include MAPKs and CKII, primarily because bioinformatic and biochemical analysis suggested their direct involvement in the phosphorylation of cellulase-regulating transcription factors [15, 16, 19].

Mitogen-activated protein kinases are ubiquitous protein kinases in eukaryotes that play the role of signal transduction and amplification [20]. It is a key component of a MAPKKK–MAPKK–MAPK signal relay system. In this signal transduction cascade, MAPKKK (MAP kinase kinase kinase) is first phosphorylated upon activation, which phosphorylates and activates MAPKK (MAP kinase kinase), which in turn phosphorylates and activates MAPK for downstream functions [21]. Six major MAPK pathways are present in the model fungus *Saccharomyces cerevisiae*, the homologues of three of which have been widely found in filamentous fungi: Hog1-like MAPK pathway, Slt2-like MAPK pathway, and Fus3-like MAPK pathway [19, 22]. In *S. cerevisiae*, the Hog1 pathway responds to high osmolarity signals and functions in combating this environmental stress, the Slt2 pathway primarily functions in cell wall integrity maintenance, and the Fus3 pathway functions in the pheromone-induced mating type differentiation process [21, 23].

Mitogen-activated protein kinases are involved in a variety of physiological functions in filamentous fungi. Hog1 homologues in filamentous fungi function primarily in high osmolarity resistance similarly with *S. cerevisiae* [24–29]. Involvement of Hog1 homologues in other processes such as sporulation [24, 27], stress response [28, 30], and secondary metabolism [27] has also been reported. Like in *S. cerevisiae*, Slt2 homologues in filamentous fungi are also primarily involved in cell wall integrity maintenance with a few exceptions [31–42]. Previous reports also showed the involvement of Slt2 in a series of other processes such as virulence [31, 32, 41], sporulation [32, 36, 37, 42], female fertility, secondary metabolism [37, 40], hyphal polarity maintenance [37, 38, 43], surface hydrophobicity maintenance [39, 41], stress response [38] and circadian rhythm maintenance [44]. The function of Fus3 homologues in filamentous fungi is quite diverse, with reported participation in pathogenicity [45–50], sporulation [48, 51], hyphal development [52–55], secondary metabolism [55], and stress response [51]. In particular, Fus3 homologues in *Trichoderma* species were shown to participate in the production of mycoparasitism-related proteins such as chitinases and *N*-acetylglucosaminidases [49, 56–58], and in the production of cellulases [49]. This function raises curiosity on whether Fus3 homologues may function in regulating cellulase expression and secretion in *T. reesei*.

Three MAPKs have been identified from in silico analysis in *T. reesei*, namely, the Fus3-like Tmk1, the Slt2-like Tmk2, and the Hog1-like Tmk3 [19]. Previous research carried out in our laboratory has shown the physiological functions of Tmk2 and Tmk3 [15, 59]. Our results showed that both Tmk2 and Tmk3 are involved in cell wall integrity maintenance. Tmk3 also participates in high osmolarity resistance and biosynthesis, while Tmk2 also participates in sporulation. The deletion of *tmk3* leads to a strong reduction of cellulase formation and transcription of cellulase-coding genes, suggesting the role of Tmk3 in positively regulating the expression of cellulase-coding genes [15]. On the contrary, the deletion of *tmk2* has little effect on the transcriptional level of cellulase-coding genes but leads to a higher cellulase formation level [59]. The role of Tmk1 in *T. reesei* was still unknown.

In this work, we aim to generate a full picture on the role of MAPKs in cellulase formation in *T. reesei*. This was done by constructing and characterizing *T. reesei Δtmk1*, thereby finding out whether Tmk1 is involved in the regulation of cellulase formation, as well as the overexpression of *tmk3* in a *T. reesei Δtmk2* strain, which led to the identification of the mechanism on how Tmk2 influences cellulase formation. Based on this functional characterization, previously identified roles of Tmk2 and Tmk3 in *T. reesei*, as well as the mechanistic investigations carried out in this work on how MAPKs influence cellulase formation in *T. reesei*, this picture of the involvement of MAPKs in cellulase production can be obtained.

## Results

### Deletion of *tmk1* in *T. reesei* leads to improved growth and cellulase formation

**Fig. 1:**
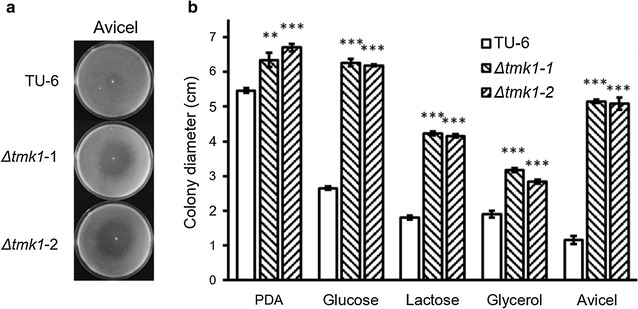
Growth of *T. reesei* TU-6 and *tmk1* deletion strains on agar plates. **a** photographs of *T. reesei* colonies on double layer Avicel agar plates (Avicel); **b** comparison of colony diameters between *T. reesei* TU-6 and *tmk1* deletion strains. TU-6, *T. reesei* TU-6; *Δtmk1*-1, *T. reesei Δtmk1*-1; *Δtmk1*-2, *T. reesei Δtmk1*-2. **p < 0.01; ***p < 0.001. *Error bar* standard deviation of three replicates

The deletion of *tmk1* in *T. reesei* was successfully carried out with homologous recombination (see Additional file 1), generating two parallel *tmk1* deletion strains, respectively, named *T. reesei Δtmk1*-1 and *T. reesei Δtmk1*-2. The parent *T. reesei* TU-6 strain and the *tmk1* deletion strains (*T. reesei Δtmk1*-1 and *T. reesei Δtmk1*-2) were grown on potato dextrose agar (PDA) plates, minimal media plates containing glycerol, glucose, or lactose as the substrate, and Avicel double layer plates (Fig. 1a). It can be observed that the two *tmk1* deletion strains form larger colonies in comparison with the parent strain. This observation is confirmed by measuring the colony diameters after 6 days of growth on Avicel double layer plates and 3 days of growth on all other plates (Fig. 1b). Without exception, both Δ*tmk1* strains grow significantly better than the parent strain. Interestingly, a larger clear zone was formed in the Avicel double layer plates on which either *tmk1* deletion strain was grown in comparison with the *T. reesei* TU-6 parent strain (Fig. 1a). These results clearly lead to the suggestion that the deletion of *tmk1* leads to improved growth and cellulase formation in *T. reesei.* No significant impact on cell wall integrity was observed after the deletion of *tmk1* (see Additional file 2).

**Fig. 2:**
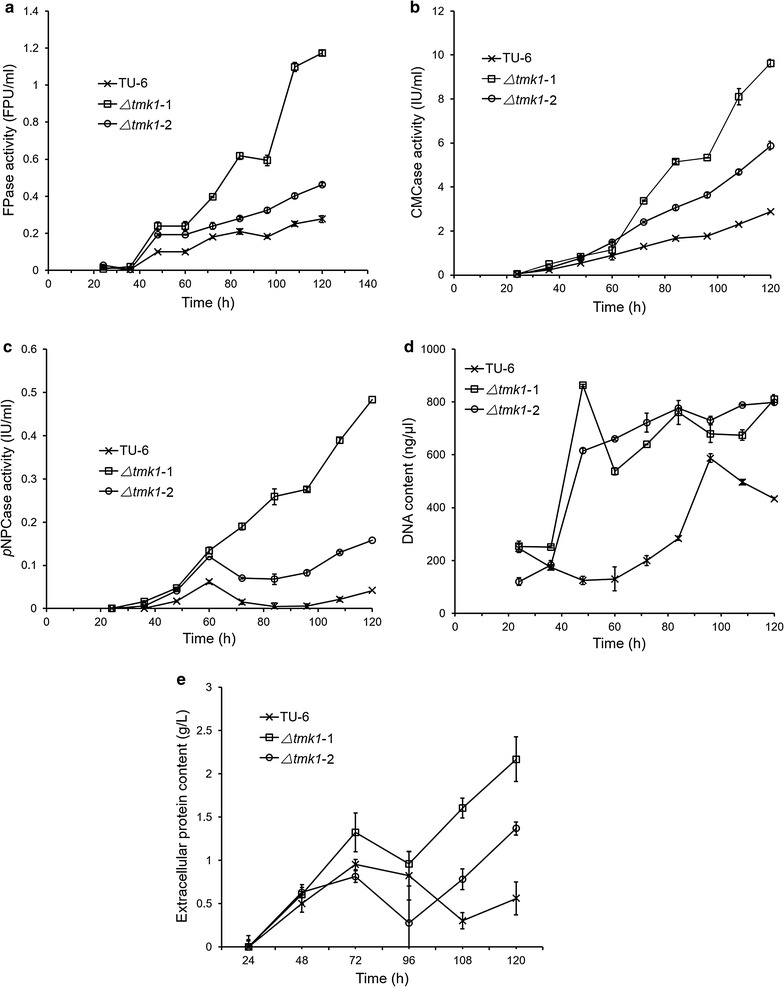
Production of cellulase activities by *T. reesei* TU-6, *Δtmk1*-1, and *Δtmk1*-2 in 3-L fermenters. **a** Production of filter paperase activity (FPase activity); **b** production of CMCase activity; **c** production of *p*NPCase activity; **d** biomass determination represented by A_260_; **e** total extracellular protein content. TU-6, *T. reesei* TU-6; *Δtmk1*-1, *T. reesei Δtmk1*-1; *Δtmk1*-2, *T. reesei Δtmk1*-2. *Error bar* standard deviation of three replicates

**Fig. 3:**
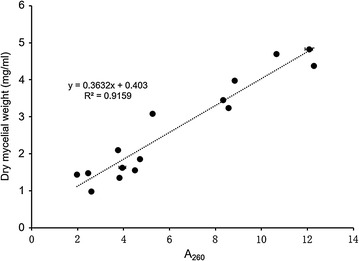
Correlation between A_260_ and dry mycelial weight

Growth and production analysis of *T. reesei* TU-6, *T. reesei Δtmk1*-1, and *T. reesei Δtmk1*-2 grown in 3 L fermenters showed the same phenomenon (Fig. 2). The overall filter paperase activities (FPase activity, FPA), CMCase activities that represent endoglucanase activities, *p*NPCase activities that represent cellobiohydrolase activities and total extracellular protein contents are all significantly improved in *tmk1* deletion strains *T. reesei Δtmk1*-1 and *T. reesei Δtmk1*-2 in comparison with the parent strain (Fig. 2a–c, e). For determination of biomass of these strains, we used a previously reported method by which total intracellular A_260_ was used as a measure for biomass [15, 60]. This approach was validated by plotting intracellular A_260_ of different *T. reesei* strains grown under different growth conditions against respective dry mycelial weight (Fig. 3), confirming the use of total intracellular A_260_ as an applicable method for biomass determination. A significant improvement of total fungal biomass in the *tmk1* deletion strains was observed with this method (Fig. 2d).

### *Tmk1* is not involved in regulation of cellulase transcription

**Fig. 4:**
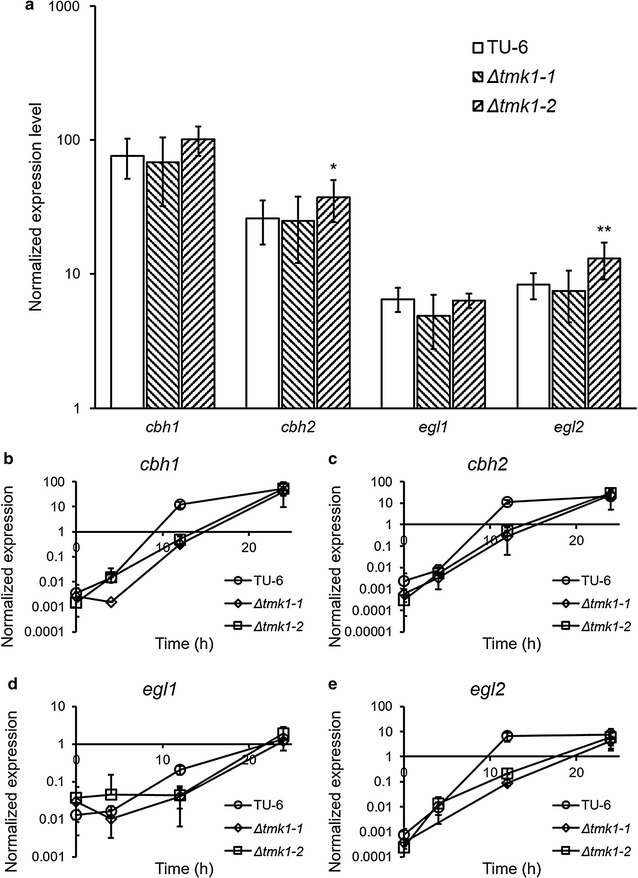
Transcriptional comparison of cellulase-coding genes between *T. reesei* TU-6, *Δtmk1*-1, and *Δtmk1*-2. **a** Expression levels 3 days after inoculation to Avicel- and wheat bran-containing media; **b** *cbh1* expression levels after transfer to Avicel- and wheat bran-containing media; **c** *cbh2* expression levels after transfer to Avicel- and wheat bran-containing media; **d** *egl1* expression levels after transfer to Avicel- and wheat bran-containing media; **e** *egl2* expression levels after transfer to Avicel- and wheat bran-containing media. Expression levels are normalized to the expression level of the actin-coding gene. TU-6, *T. reesei* TU-6; *Δtmk1*-1, *T. reesei Δtmk1*-1; *Δtmk1*-2, *T. reesei Δtmk1*-2. *p < 0.05; **p < 0.01. *Error bar* standard deviation of nine replicates

Despite our observation that cellulase production is improved following *tmk1* deletion, transcriptional analysis of major cellulase-coding genes *cbh1*, *cbh2*, *egl1*, and *egl2* after 3 days of growth using Avicel and wheat bran as the carbon source showed little difference between the parent TU-6 strain and the two *tmk1* deletion strains *Δtmk1*-1 and *Δtmk1*-2 (Fig. 4a). Change of transcriptional levels of the same genes in the first 24 h after the transfer from glycerol-containing media to Avicel- and wheat bran-containing media showed similar results (Fig. 4b–e). A suggestion can be made from these results that Tmk1 is not involved in the regulation of cellulase transcription in *T. reesei*. The reason for the improved cellulase formation in *tmk1* deletion strains should therefore be the improved growth of *T. reesei tmk1* deletion strains, rather than the specific regulation of Tmk1 on the expression of cellulase-coding genes. A further suggestion can be made from these results that Tmk1 is not involved in the signal transduction pathway transmitting cellulase induction cellulose signals.

### Damage of cell wall integrity by the deletion of *tmk2* and reinforcing cell wall integrity by overexpressing *tmk3*

**Fig. 5:**
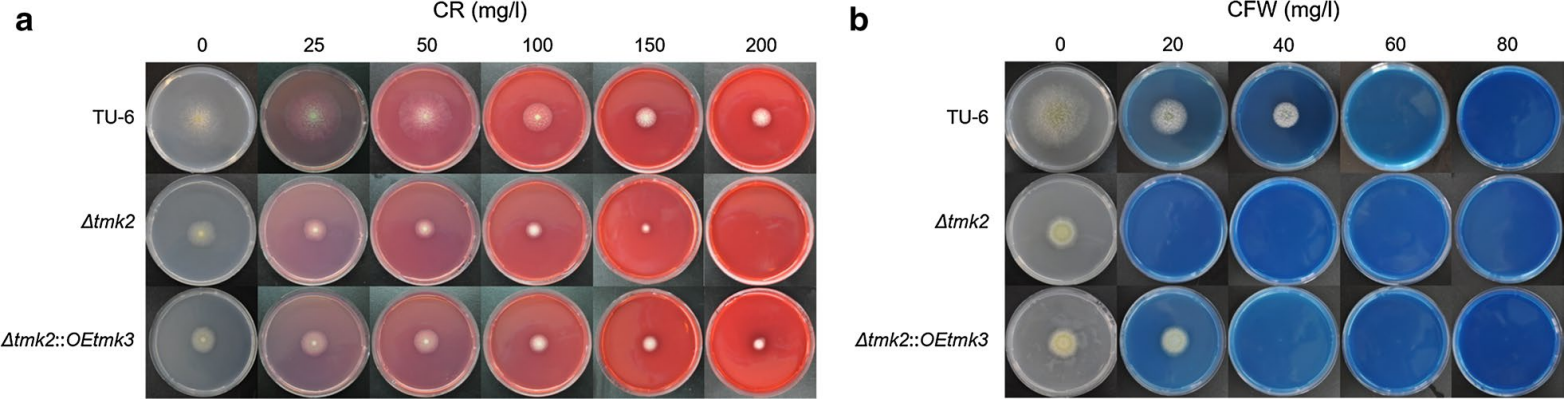
The sensitivity of *T. reesei* TU-6, *Δtmk2* and *Δtmk2::OEtmk3* to CR and CFW. **a** Growth of *T. reesei* TU-6, *T. reesei Δtmk2* and *T. reesei Δtmk2::OEtmk3* on CR-containing agar plates; **b** growth of *T. reesei* TU-6, *T. reesei Δtmk2* and *T. reesei Δtmk2::OEtmk3* on CFW-containing agar plates. TU-6, *T. reesei* TU-6; *Δtmk2*, *T. reesei Δtmk2*; *Δtmk2::OEtmk3*, *T. reesei Δtmk2::OEtmk3*

Both Tmk2 and Tmk3 were shown to participate in cell wall integrity maintenance, and the deletion of either *tmk2* or *tmk3* led to damaged cell wall integrity [15, 59]. They may therefore compensate each other’s function in cell wall integrity maintenance if one gene is deleted. In this work, we overexpressed *tmk3* using the previously constructed *T. reesei Δtmk2* strain [59] as the parent strain to improve cell wall integrity from *T. reesei Δtmk2* (see Additional file 3). By replacing *tmk3* promoter with a strong P*gpd* promoter, we were able to increase *tmk3* transcription by 11.80-folds (*p* = 1.82 × 10^−10^, *n* = 9). Sensitivity assays to CR and CFW were carried out for *T. reesei* TU-6, *T. reesei Δtmk2*, and *T. reesei Δtmk2*::*OEtmk3* (Fig. 5). *T. reesei* TU-6 was able to tolerate 200 mg L^−1^ CR and 20 mg L^−1^ CFW, while *T. reesei Δtmk2* cannot, suggesting that tolerance to CR and CFW is reduced in *T. reesei Δtmk2* in comparison with the parent *T. reesei* TU-6 strain, in agreement with the previous report [59]. By overexpressing *tmk3*, this tolerance is regained, suggesting restoration of cell wall integrity in comparison with *T. reesei Δtmk2* (Fig. 5).

### Cell wall integrity negatively impacts cellulase secretion in *T. reesei* TU-6, *T. reesei Δtmk2*, and *T. reesei Δtmk2::OEtmk3*

**Fig. 6:**
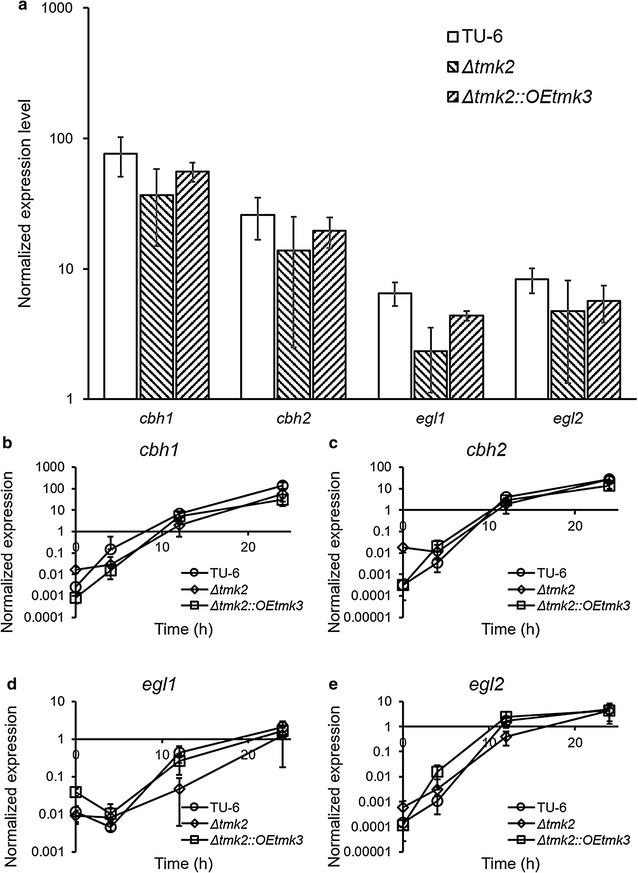
Transcriptional comparison of cellulase-coding genes between *T. reesei* TU-6, *Δtmk2* and *Δtmk2::OEtmk3*. **a** Expression levels 3 days after inoculation to Avicel- and wheat bran-containing media; **b** *cbh1* expression levels after transfer to Avicel- and wheat bran-containing media; **c** *cbh2* expression levels after transfer to Avicel- and wheat bran-containing media; **d** *egl1* expression levels after transfer to Avicel- and wheat bran-containing media; **e** *egl2* expression levels after transfer to Avicel- and wheat bran-containing media. Expression levels are normalized to the expression level of the actin-coding gene. TU-6, *T. reesei* TU-6; *Δtmk2*, *T. reesei Δtmk2*; *Δtmk2::OEtmk3*, *T. reesei Δtmk2::OEtmk3*. *Error bar* standard deviation of nine replicates

The transcription of cellulase-coding genes *cbh1*, *cbh2*, *egl1,* and *egl2* was compared between *T. reesei* TU-6, *T. reesei Δtmk2 and T. reesei Δtmk2::OEtmk3* grown using Avicel and wheat bran as the carbon source for 3 days (Fig. 6a). Although a slight difference can be seen between the three strains, the difference is too small to be meaningful. This finding is confirmed by the similar transcriptional levels of the same cellulase-coding genes in the three strains in the first 24 h after the carbon source changed from glycerol to Avicel and wheat bran (Fig. 6b–e). From these transcriptional analyses, suggestions can be made that the deletion of *tmk2* and subsequent overexpression of *tmk3* do not lead to changes in the transcriptional levels of cellulase-coding genes, in agreement with the previous reports [59].

**Fig. 7:**
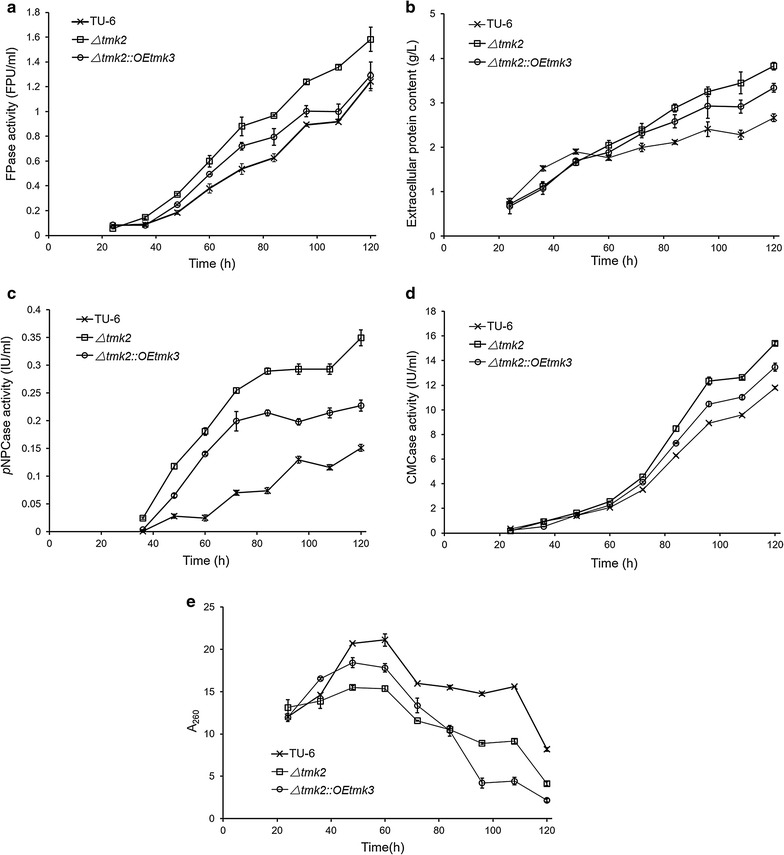
Production of cellulase by *T. reesei* TU-6, *Δtmk2*, and *Δtmk2::OEtmk3* in 3-L fermenters. **a** Production of filter paperase activity (FPase activity); **b** production of extracellular proteins; **c** production of *p*NPCase activity; **d** production of CMCase activity; **e** biomass determination represented by A_260_. TU-6, *T. reesei* TU-6; *Δtmk2*, *T. reesei Δtmk2*; *Δtmk2::OEtmk3*, *T. reesei Δtmk2::OEtmk3*. *Error bar* standard deviation of three replicates

Despite the lack of transcriptional changes in cellulase-coding genes, significant differences can be observed between cellulase production levels of the three strains in 3 L fermenters (Fig. 7). Although the biomass represented by volumetric A_260_ in the cultures decreased in *T. reesei Δtmk2* in comparison with *T. reesei* TU-6 (Fig. 7e), the FPA, *p*NPCase, CMCase activities, and the extracellular protein content increased significantly in *T. reesei Δtmk2* strain (Fig. 7a–d). This is in consistence with the previous reports that *T. reesei Δtmk2* secrets more cellulases than the parent strain, despite no change in transcription of cellulase-coding genes is induced by *tmk2* deletion [59]. Following the overexpression of *tmk3*, the total biomass remained largely similar in comparison with *T. reesei Δtmk2* (Fig. 7e). The FPA, *p*NPCase, and CMCase activities, however, significantly decreased over *T. reesei Δtmk2* (Fig. 7a, c, d). The same decrease is observed for extracellular protein content (Fig. 7b). A suggestion can be therefore made that the decrease of cell wall integrity by deleting *tmk2* leads to improved cellulase production, while further increase of cell wall integrity by overexpressing *tmk3* leads to decreased cellulase production.

**Fig. 8:**
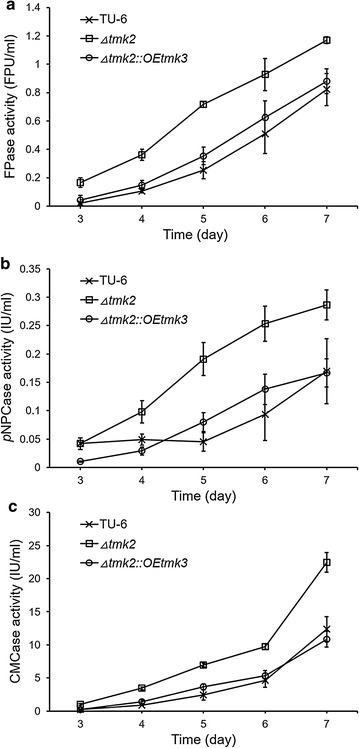
Production of cellulase by *T. reesei* TU-6, *Δtmk2,* and *Δtmk2::OEtmk3* in shake flask experiments. **a** Production of filter paperase activity (FPase activity); **b** production of *p*NPCase activity; **c** production of CMCase activity. TU-6, *T. reesei* TU-6; *Δtmk2*, *T. reesei Δtmk2*; *Δtmk2::OEtmk3*, *T. reesei Δtmk2::OEtmk3*. *Error bar* standard deviation of three replicates

This observation of the relationship between cell wall integrity and cellulase secretion is confirmed by shake flask experiments assaying secretion of cellulase activities in *T. reesei* TU-6, *T. reesei Δtmk2*, and *T. reesei Δtmk2::OEtmk3* (Fig. 8). *T. reesei Δtmk2* that has a weaker cell wall secretes more cellulase activities than the parent *T. reesei* TU-6 strain, while decreased cellulase activities were observed for *T. reesei Δtmk2::OEtmk3* whose cell wall integrity is partially restored.

## Discussion

The heterologous nature of *T. reesei* cells and the very complex mechanism of cellulase regulation have prevented us from fully elucidating the mechanisms for cellulase induction and repression by carbon sources. An example of the level of complexity of this regulation is the cross-regulation of transcription factors. As was reported previously with very delicate transcriptional analysis, full expression of the cellulase-inducing transcription factors Xyr1 and ACEII requires the presence of the cellulase repressing transcription factor Cre1 [61]. This seemingly aberrant phenomenon is actually a great testimony of how cellulase expression is fine-tuned by transcription factors in response to external signals. For this very reason, investigating the signal transduction pathways has the benefit of avoiding the complex cross-regulation between transcription factors, and can serve as an entry point for elucidating the complete cellulase induction and repression pathways, because the direct targets and upstream factors of a signal transduction pathway whose function is defined should participate in the same function.

Mitogen-activated protein kinase pathways are the best characterized signal transduction pathways in eukaryotes. Although the functions of many MAPK pathways are conserved in fungi, they also play specific roles for each species. Take Hog1-like pathways for instance, its role in high osmolarity resistance is universally conserved in fungi [24, 26], but the role in cellulase production is not reported in any fungal species except for *T. reesei* [15]. In *Trichoderma* species, the Fus3-like pathways are involved in mycoparasitism [49, 56–58]. The ability for mycoparasitism in *T. reesei* is, however, significantly reduced during evolution [62]. Since the function of mycoparasitism requires secretion of hydrolases such as chitinases and *N*-acetylglucosaminidases [49, 56, 57], we originally expected that during evolution, accompanying the reduction of mycoparasitism functions, *T. reesei* could use the Fus3-like Tmk1 to regulate the expression of cellulases which are also extracellular hydrolases. This, however, is not the case, and the role of Tmk1 on cellulase formation is limited to the growth rate-dependent production level, rather than signal transduction and transcription levels.

Both Tmk2 and Tmk3 pathways are involved in cell wall integrity maintenance in *T. reesei* [15, 59]. The primary cell wall integrity related MAPK conserved in fungi is the Slt2-like MAPK. The influence of Slt2-like Tmk2 on cellulase formation in *T. reesei* is found to be related to cell wall integrity in this work: Tmk2 maintains cell wall integrity, and negatively impacts cellulase secretion because of the intact cell wall. Indeed, the enhancement of protein secretion by damaging cell walls has been shown for other proteins [63], although reports on the relationship between cell wall integrity and cellulase secretion are rare. Suggestions can therefore be made that by hampering cell wall integrity, the secretion of cellulases is made easy because the enzymes now have an easier time penetrating cell wall, therefore leading to the promotion of cellulase formation.

Mechanistic investigations carried out in this and previous work lead to a suggestion that Tmk3 is the only MAPK that is involved in the transduction of cellulase-inducing signal [15, 59]. Recently, a phosphoproteomic investigation of *T. reesei* exposed to sophorose and distiller’s spent grain showed that Tmk3 is phosphorylated under these cellulase-inducing conditions [64]. What’s particularly interesting is that Tmk3 was also the only MAPK that is found to be phosphorylated when exposed to sophorose or distiller’s spent grain. These proteomic data are in full consistent with our finding, and supports our conclusion made in this work. We believe these findings could be a starting point on elucidating the transduction pathways of cellulase-inducing signals in *T. reesei*, with further work focused on finding out factors and pathways upstream and downstream of Tmk3.

Although Tmk1 and Tmk2 are not involved in signal transduction pathways leading to the induction or repression of cellulase expression, they can both potentially be used in engineering *T. reesei* for better cellulase titers because cellulase production is improved by deletion either gene even if growth was damaged for the case of *tmk2*. Conversely, although Tmk3 is involved in the transduction of cellulase induction signals, overexpressing this gene does not further improve transcription of cellulase-coding genes (Fig. 6), making it a bad target for engineering. This phenomenon gives Tmk1 and Tmk2 more practical/industrial values, although more focus should be given to Tmk3 on a microbial physiology perspective.

## Conclusions

In conclusion, in this work, the role of MAPKs in *T. reesei* on cellulase formation was investigated. Two primary conclusions can be drawn from this work: Tmk1 is not involved in the regulation of cellulase-coding genes but represses cellulase formation by repressing cellular growth; Tmk2 is not involved in regulating the transcription of cellulase-coding genes either, but its role in maintaining cell wall integrity hampers cellulase secretion. The role of cell wall integrity in deterring cellulase formation, as strongly suggested by the hampered cellulase secretion in the cell wall integrity restored *T. reesei Δtmk2::OEtmk3* strain, is a particularly interesting finding.

**Fig. 9:**
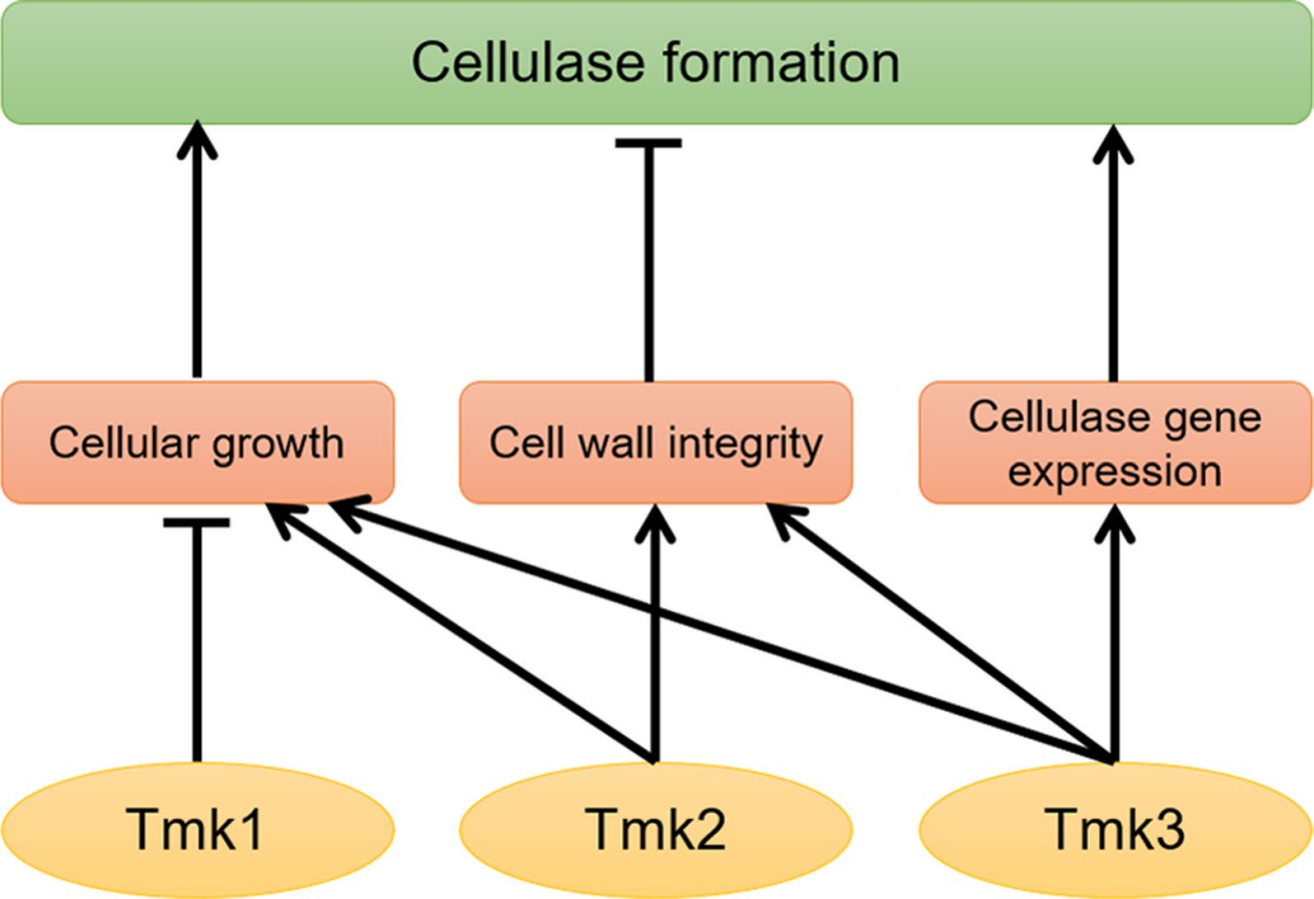
Involvement of *T. reesei* MAPKs in cellulase formation

Results obtained from this and previous [15, 59] work lead to the answer to an important question: Which MAPK in *T. reesei* is involved in cellulase expression and production, and how? A full picture of the role of MAPKs on cellulase formation in *T. reesei* can be generated based on these investigations (Fig. 9). In this tentative model, Tmk3 stands out as the only MAPK involved in signal transduction pathways leading to the induction of cellulases, while Tmk1 and Tmk2 are both involved in repressing cellulase formation, although not by altering transcription of cellulase-coding genes. These investigations contribute to further understanding of the cellulase-regulating signal transduction processes in *T. reesei*, and contribute to the elucidation of cellulase regulation mechanisms in this key industrial cellulase-producing filamentous fungus. Furthermore and most importantly, findings from this work contribute to identifying appropriate targets for engineering *T. reesei* for better cellulase production, which is the key for biofuel production from lignocelluloics.

## Methods

### Strains

The non-homologous end joining pathway-deficient, uridine auxotrophic defective *T. reesei* TU-6 (ATCC MYA-256) *Δku70* strain was used as the parent strain in this work, and hereafter abbreviated as *T. reesei* TU-6 (or TU-6) strain. *T. reesei Δtmk2* strain is the *tmk2* deletion strain using *T. reesei* TU-6 as the parent strain [59].

### Construction of *T. reesei Δtmk1* and *T. reesei Δtmk2::OEtmk3* strains

The construction of *T. reesei Δtmk1* and *T. reesei Δtmk2::OEtmk3* strains was carried out by homologous recombination essentially as previously described [65]. For construction of *T. reesei Δtmk1*, transformed DNA fragment contains 1.8 kb upstream homologous arm, *pyr4* that restores uridine biosynthesis, and 1.8 kb downstream homologous arm (see Additional file 1). *T. reesei* TU-6 was used as the parent strain for *tmk1* deletion. For construction of *T. reesei Δtmk2::OEtmk3*, transformed DNA fragment contains a 2 kb upstream homologous arm (3500 bp to 1500 bp upstream of *tmk3*), P*gpd* from *Aspergillus nidulans*, *ptrA* gene from *Aspergillus oryzae* that confers pyrithiamine resistance and *tmk3* sequence as the downstream homologous arm (2.2 kb) (see Additional file 3). *T. reesei Δtmk2* was used as the parent strain for *tmk3* overexpression. Two parallel *tmk1* deletion strains *T. reesei Δtmk1*-1 and *T. reesei Δtmk1*-2 were generated.

### Southern blotting analysis

Analysis of genotypes of *T. reesei* strains was carried out by Southern blotting following the previously published protocols [15]. For the analysis of *T. reesei Δtmk1* genome, *Kpn*I was used as the restriction digestion enzyme for genome digestion. For the analysis of *T. reesei Δtmk2::OEtmk3* genome, *Hae*II was used as the restriction digestion enzyme for genome digestion.

### Growth of *T. reesei* strains

Minimal media (MM) solution used for media preparation was prepared following the previously reported recipe [15]. For growth in shake flask in transcription abundance determination experiments, growth media contain MM solution, 2% Avicel, and 2% wheat bran. For growth in shake flask for genome extraction and Southern blotting, growth media contain MM solution and 2% glucose. For growth on plates, media contain MM solution, 2% agarose and 2% glucose, 2% lactose, or 2% glycerol. PDA plates contain 20% potato syrup, 2% agarose, and 2% sucrose. Double layer Avicel agar plates were prepared as previously described [15]. All plates and media contain 0.1% uridine. Congo Red (CR)- and Calcofluor White (CFW)-containing agar plates were prepared by adding CR or CFW to PDA plates. All cultures or plates were incubated at 30 °C for growth. Shake flask cultures were shaken at 200 rpm during cultivation. All parallel growth were carried out under the same illumination conditions.

For fermentation experiment, seed cultures were prepared by growing *T. reesei* strains in MM solution containing 2% Avicel and 2% wheat bran for 48 h (1 × 10^7^ spore inoculation). Fermentation was carried out in 3-L fermenters (Model BLB10-3GJG, Bailun Bio-Technology Co., Ltd., Shanghai, China) containing 2 L of media (MM solution, 2% Avicel, 2% wheat bran). Two hundred milliliters of seed cultures were inoculated to initiate fermentation. Growth took place for 120 h at 30 °C. Agitation rate was adjusted to allow dissolved oxygen levels above 30%.

### Phenotypic analysis

Phenotypic analysis of *T. reesei Δtmk1* strains was carried out by inoculating 1 × 10^5^ spores onto plates and grown for 3 days (6 days for double layer Avicel agar plates) prior to colony diameter determination. Three individual replicates were carried out.

### Biochemical analysis

The analysis of A_260_, FPase (Filter Paperase) activities, *p*NPCase (*p*-nitrophenyl-β-d-cellobiosidase) activities, CMCase (carboxymethyl cellulase) activities, and protein concentration determination with the Lowry assay were carried out following the previously described protocols [15, 66, 67].

The correlation between A_260_ and dry mycelial weight was determined by first cultivating *T. reesei* TU-6 and *T. reesei* TU-6 *Δtmk3* in potato dextrose broth (PDA minus agar) and MM media with glucose or lactose as the carbon sources in 3-L fermenters. For dry mycelial weight determination, approximately 11 mL of the cultures were extracted from fermenters, washed, filtered, and dried in an oven at 80 °C for over 12 h. The mycelia were subsequently weighed. The determination of A_260_ was carried out as previously described [15]. Four replicates were carried out.

### CR and CFW resistance

For analysis of CR and CFW resistance, approximately 1 × 10^5^ spores of *T. reesei* strains were inoculated on CR- and CFW-containing plates and were grown at 30 °C for 3 days prior to colony diameter determination. Three replicates were carried out.

### qPCR analysis

For qPCR analysis, approximately 1 × 10^6^ spores of *T. reesei* strains were inoculated to media containing 2% Avicel and 2% wheat bran and grown for 3 days prior to extraction of total RNA for transcription quantification. The qPCR procedures and data manipulation were carried out essentially as previously described [15]. Three biological replicates and three technical replicates for each biological replicate were carried out.

For determination of transcriptional abundance of cellulase-coding genes at different time points after inoculation to media containing Avicel + wheat bran, spores were first inoculated to MM media +2% glucose and grew for 2 days, after which mycelia were extracted, washed, and inoculated to MM media +2% glycerol for growth for 1 day. The mycelia were further washed and inoculated to MM media containing 2% wheat bran and 2% Avicel. Cells were extracted for transcriptional analysis upon transfer, as well as 4, 12, and 24 h after the transfer.

### Statistics

To test if two sets of data are statistically different, two-tailed Student’s *t*-tests were carried out. *p* < 0.05 was considered statistically significant. A minimum of three replicates were carried out for each experiment.

## Additional files

**Additional file 1.** Southern blotting analysis of *T. reesei* TU-6, *Δtmk1*-1 and *Δtmk1*-2. Panel A: Schematic drawing of strain construction and southern blotting analysis. Panel B: southern blotting of *T. reesei* strains. M: DNA marker; TU-6: *T. reesei* TU-6; *Δtmk1*-1: *T. reesei Δtmk1*-1; *Δtmk1*-2: *T. reesei Δtmk1*-2.

**Additional file 2.** The sensitivity of *T. reesei* TU-6, *Δtmk1*-1 and *Δtmk1*-2 to CR and CFW. Panel A: growth of *T. reesei* TU-6, *T. reesei Δtmk1*-1 and *T. reesei Δtmk1*-2 on CR-containing agar plates; Panel B: growth of *T. reesei* TU-6, *T. reesei Δtmk1*-1 and *T. reesei Δtmk1*-2 on CFW-containing agar plates. TU-6, *T. reesei* TU-6; *Δtmk1*-1, *T. reesei Δtmk1*-1; *Δtmk1*-2, *T. reesei Δtmk1*-2.

**Additional file 3.** Southern blotting analysis of *T. reesei* TU-6, *Δtmk2* and *Δtmk2::OEtmk3*. Panel A: Schematic drawing of strain construction and southern blotting analysis. Panel B: southern blotting of *T. reesei* strains. M: DNA marker; *Δtmk2*: *T. reesei Δtmk2*; *Δtmk2::OEtmk3*: *T. reesei Δtmk2::OEtmk3.*
