# Supplementary material for: Role of Trichoderma reesei mitogen-activated protein kinases (MAPKs) in cellulase formation
Source: Biotechnol Biofuels. 2017 Apr 20;10:99. doi: 10.1186/s13068-017-0789-x (PMC5397809; doi:10.1186/s13068-017-0789-x)

Additional File 1. Southern blotting analysis of *T. reesei* TU-6, *Δtmk1*-1 and *Δtmk1*-2. Panel A: Schematic drawing of strain construction and southern blotting analysis. Panel B: southern blotting of *T. reesei* strains. M: DNA marker; TU-6: *T. reesei* TU-6; *Δtmk1*-1: *T. reesei Δtmk1*-1; *Δtmk1*-2: *T. reesei Δtmk1*-2.

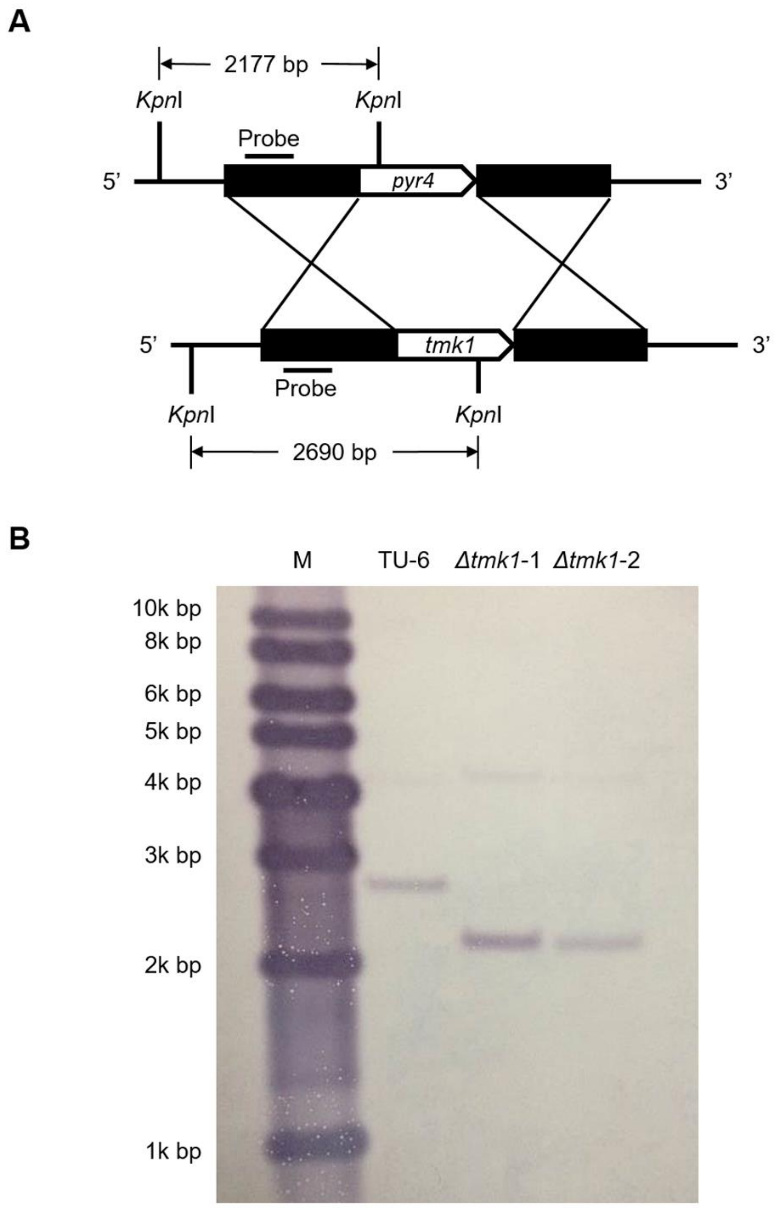

Supplement: Supplementary file 1 — Additional file 1. Southern blotting analysis of T. reesei TU-6, Δtmk1-1 and Δtmk1-2. Panel A: Schematic drawing of strain construction and southern blotting analysis. Panel B: southern blotting of T. reesei strains. M: DNA marker; TU-6: T. reesei TU-6; Δtmk1-1: T. reesei Δtmk1-1; Δtmk1-2: T. reesei Δtmk1-2. [file 13068_2017_789_MOESM1_ESM.pdf]
