# Supplementary material for: Role of Trichoderma reesei mitogen-activated protein kinases (MAPKs) in cellulase formation
Source: Biotechnol Biofuels. 2017 Apr 20;10:99. doi: 10.1186/s13068-017-0789-x (PMC5397809; doi:10.1186/s13068-017-0789-x)

Additional File 2. The sensitivity of *T. reesei* TU-6, *Δtmk1*-1 and *Δtmk1*-2 to CR and CFW. Panel A: growth of *T. reesei* TU-6, *T. reesei Δtmk1*-1 and *T. reesei Δtmk1*-2 on CR-containing agar plates; Panel B: growth of *T. reesei* TU-6, *T. reesei Δtmk1*-1 and *T. reesei Δtmk1*-2 on CFW-containing agar plates. TU-6, *T. reesei* TU-6; *Δtmk1*-1, *T. reesei Δtmk1*-1; *Δtmk1*-2, *T. reesei Δtmk1*-2.

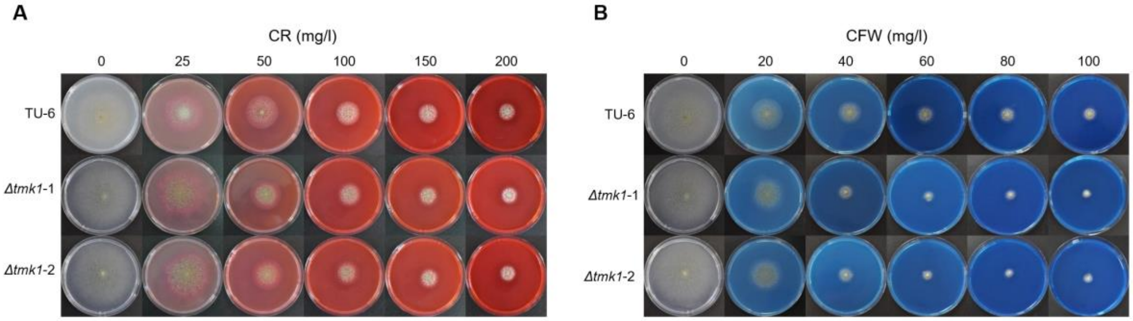

Supplement: Supplementary file 2 — Additional file 2. The sensitivity of T. reesei TU-6, Δtmk1-1 and Δtmk1-2 to CR and CFW. Panel A: growth of T. reesei TU-6, T. reesei Δtmk1-1 and T. reesei Δtmk1-2 on CR-containing agar plates; Panel B: growth of T. reesei TU-6, T. reesei Δtmk1-1 and T. reesei Δtmk1-2 on CFW-containing agar plates. TU-6, T. reesei TU-6; Δtmk1-1, T. reesei Δtmk1-1; Δtmk1-2, T. reesei Δtmk1-2. [file 13068_2017_789_MOESM2_ESM.pdf]
