# Supplementary material for: Role of Trichoderma reesei mitogen-activated protein kinases (MAPKs) in cellulase formation
Source: Biotechnol Biofuels. 2017 Apr 20;10:99. doi: 10.1186/s13068-017-0789-x (PMC5397809; doi:10.1186/s13068-017-0789-x)

Additional File 3. Southern blotting analysis of *T. reesei* TU-6, *Δtmk2* and *Δtmk2::OEtmk3*. Panel A: Schematic drawing of strain construction and southern blotting analysis. Panel B: southern blotting of *T. reesei* strains. M: DNA marker; *Δtmk2*: *T. reesei Δtmk2*; *Δtmk2::OEtmk3*: *T. reesei Δtmk2::OEtmk3*.

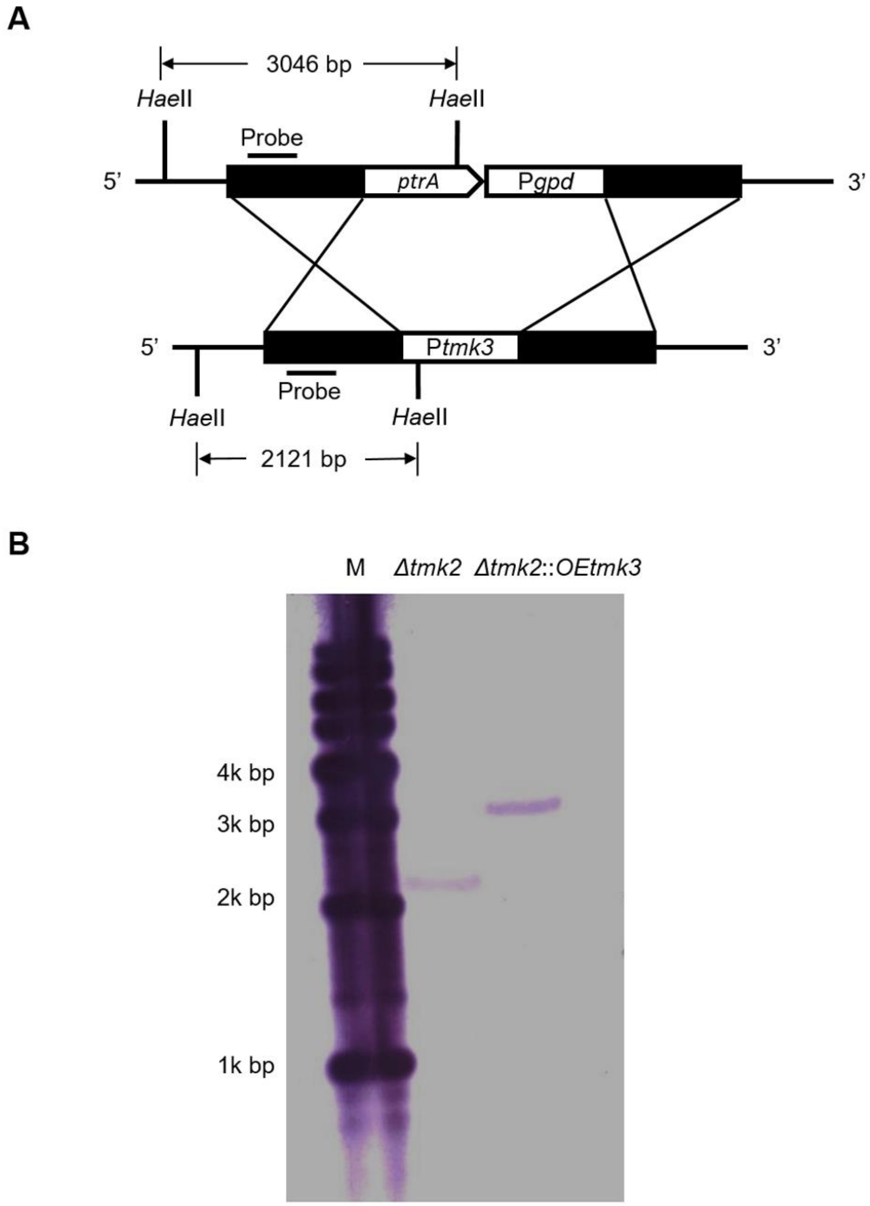

Supplement: Supplementary file 3 — Additional file 3. Southern blotting analysis of T. reesei TU-6, Δtmk2 and Δtmk2::OEtmk3. Panel A: Schematic drawing of strain construction and southern blotting analysis. Panel B: southern blotting of T. reesei strains. M: DNA marker; Δtmk2: T. reesei Δtmk2; Δtmk2::OEtmk3: T. reesei Δtmk2::OEtmk3. [file 13068_2017_789_MOESM3_ESM.pdf]
